# Does p‐lactate increase in patients with GSD1 after ingesting a meal with common‐size sources of fructose and galactose? Observations from a prospective, non‐blinded, crossover pilot study

**DOI:** 10.1002/jmd2.12457

**Published:** 2025-01-16

**Authors:** Camilla Diana B. Caroee, Allan M. Lund

**Affiliations:** ^1^ Department of Pediatrics, Center for Inherited Metabolic Diseases Copenhagen University Hospital, Rigshospitalet Copenhagen Denmark; ^2^ Department of Clinical Genetics, Center for Inherited Metabolic Diseases Copenhagen University Hospital, Rigshospitalet Copenhagen Denmark; ^3^ Department of Clinical Medicine, Faculty of Health and Medical Sciences University of Copenhagen Copenhagen Denmark

**Keywords:** diet, fructose, galactose, glucose, glycogen storage disease type 1, lactate

## Abstract

Ingestion of fructose and galactose may result in elevated lactate concentrations in patients with glycogen storage disease type 1 (GSD1). In this randomized cross‐over pilot study, 7 patients with GSD 1a (6) and GSD1b (1) orally consumed a common‐size fructose and galactose from either 200 mL of skimmed milk, 200 mL juice or 200 mL water. This was given after a night with their usual dietary treatment using either cornstarch, glycosade or continuous feed. P‐lactate and ‐glucose were measured 2 h before dosing (*T* = −120 min and −60 min). At baseline (*T* = 0), p‐lactate, p‐glucose, p‐triglycerides, p‐uric acid and p‐alanine were measured just before dosing. P‐lactate and p‐glucose were measured every 30 min for 4 h. Four hours after the consumption (*T* = 240 min, end‐of‐test), levels of p‐lactate, p‐glucose, p‐triglycerides, p‐uric acid and p‐alanine were measured. P‐lactate increased in three patients with mean of 0.3 mmol/L (range 0.2–0.6 mmol/L) after consuming milk. The highest level was seen after 60 min. A decrease was seen in three patients. P‐lactate increased in four patients with a mean increase at 1.3 mmol/L (range 0.2–2.2 mmol/L) after consuming 200 mL juice. A peak increase was seen after the first 30 min in two patients whereas the peak in the remaining two patients was at 60 min; all values decreased to baseline values after further 60 min. In two patients, p‐lactate was unchanged, respectively, decreased after juice ingestion. Calculation of galactose and fructose AUC percentage change after challenge did not reveal consistent increase or decreases.

**Synopsis**

Theoretically galactose and fructose may end up in lactate. Earlier studies support this when the substrates are given in large amounts, whereas the effect of the smaller amounts used in this pilot study is less convincing. Thus, there is a need for further research to study this in more detail and especially when giving substrates in a quantity similar to that found in a normal meal.

## 1 INTRODUCTION

Glycogen storage disease type 1 (GSD1) is an inborn error of carbohydrate metabolism. Two subtypes have been identified; GSD type 1a caused by glucose‐6‐phosphatase deficiency and GSD type 1b caused by glucose‐6‐phosphate translocase deficiency.

Impaired glucose‐6‐phosphatase activity is crucial for both glycogenolysis and gluconeogenesis, and the enzyme deficiency causes insufficient endogenous glucose production and glycogen accumulation in liver, kidney and intestinal mucosa.^1^, ^2^, ^3^ The biochemical hallmarks of GSD1 include severe hypoglycemia after a short period of fasting as soon as exogenous glucose supply has been used. In addition, hyperlactataemia, hyperlipidemia and raised uric acid as well as slight increases in liver transaminases are seen.^4^

Daily dietary management includes provision of an amount of glucose that mirrors the normal liver glucose production rate, using mainly complex carbohydrates. It aims to maintain euglycemia and normal metabolite concentrations (i.e. lactate, triglycerides, cholesterol and uric acid) with the aim of preventing long‐term complications. Long‐term complications include osteopenia, gout, adenomas, which may transform into hepatocellular carcinoma, renal tubular and glomerular disease.^5^ Long‐term complications are thought to be minimized by good metabolic control, including normoglycemia and normolactataemia.^6^

Deficiency of glucose‐6‐phosphatase blocks the conversion of galactose and fructose to glucose, and these carbohydrates cannot be used to maintain normoglycemia. Instead, galactose and fructose may be converted to lactate or glycogen. Earlier research from the 1950s–1970s showed that enteral bolus ingestions of large amounts of fructose, sucrose and galactose resulted in elevated lactate concentrations.^7^, ^8^, ^9^ Opinions differ about the need to restrict fructose and galactose and the related disaccharides sucrose and lactose in the diet. In the European Collaborative Study on GSD1 including 288 patients, 62% of the patients were on a restricted lactose and fructose diet to varying degrees, and 38% were on a “free diet.”^1^ Some centers find it essential to limit the amount of such carbohydrates to less than 2.5 g/meal to optimize metabolic control.^10^

Feeding difficulties and eating disorders are seen in patients with GSD1,^11^, ^12^ and the dietary treatment is often a burden for the family affecting the QoL.^13^ Dietary restriction of simple carbohydrates such as fructose and galactose and thereby also sucrose and lactose will make limitations in allowed intake of many dairy products, fruit and vegetables making the food colorless, less appetizing and will reduce the natural intake of a great variety of vitamins and minerals.

Early studies provided patients with a large amount of carbohydrates that are not normally ingested in a single meal. In this study, we assess the short‐term consequences in patients with GSD1 when ingesting galactose and fructose in an amount comparable to what may be ingested during a normal meal.

## 2 PATIENTS AND METHODS

### 2.1 Population

This study was designed as a randomized, non‐blinded, cross‐over pilot study. Ten patients from Centre for Inherited Metabolic Diseases, Copenhagen University Hospital, Rigshospitalet (CIMD) were invited to participate in the study. Two patients refused participation due to too much hospitalization, and one had a fever at the enrollment. Except one patient with only liver biopsy‐based enzymatic confirmation of GSD1a, all included patients had a molecularly proven diagnosis of GSD 1a (6) or GSD1b (1) (Table 1). The study protocol was approved by the ethics committee (notification number 64365). All recruited subjects gave written informed consent to participate before any trial‐related procedures were performed.

**TABLE 1:** Characteristics of the study population.

	Patient 1	Patient 2	Patient 3	Patient 4	Patient 5	Patient 6	Patient 7
Type GSD	GSD 1a	GSD 1a	GSD 1b	GSD 1a	GSD 1a	GSD 1a	GSD 1a
Age (years)	14	13	5	16	23	26	34
Gender	Female	Male	Female	Male	Male	Female	Female
Weight (kg)	58.9	46.3	19.8	50.5	77.2	51.5	65.2
Genotype	c.247C<T, p.(Arg83Cys); c.562G>A, p.(Gly188Ser)	c.1039C>T, p.(Gln347X); c.230+1G>C	c.1015G>T, p.(Gly339Cys); c.1015G>A, p.(Gly339Ser)	c.508C>T, p.(Arg170X); c.508C>T, p.(Arg170X)	c.247C<T, p.(Arg83Cys) c.247C<T, p.(Arg83Cys)	Not available	c.248G>A, p.(Arg83His); c.508C>T, p.(Arg170X)
Medicine	Nil	Nil	Nil	Nil	Allopurinol	Allopurinol, Enalapril	Losartan, Allopurinol
Diet and metabolic control parameters at last out‐patient visit							
Energy (KJ)	9460	7620	5810	8428	9913	5227	9433
Carbohydrate (E%)	66	61	58	72	77	72	77
Protein (g/kg/day) (E%)	1.3 13	1.5 14	2.5 14	1.1 10	0.7 9	0.8 13	0.6 7
Fat (E%)	19	22	27	16	14	15	14
Night feed	Cornstarch	Cornstarch	Continuous glucose feed	Cornstarch	Glycosade	Cornstarch	Cornstarch
Fructose (g/day)	1.5	1.9	1.4	1.8	4.1	8.1	4.7
Galactose (g/day)	2.4	0.2	0	0.7	0	0.4	0
Lactate (mmol/L)	2.6	3.8	2.5	2.4	4.2	3.3	2.7
Glucose (mmol/L)	4.7	6.5	7.0	5.8	5.1	3.4	2.9
Triglycerides (mmol/L)	1.77	1.51	1.65	3.16	10.7	16.0	9.1
Urate (mmol/L)	0.38	0.36	0.26	0.48	0.33	0.46	0.37

### 2.2 Methods

The patients were admitted to CIMD the night before the dosing with galactose/fructose. Upon admission an intravenous line was placed. All blood samples were drawn from this line. During the night, the patients took their usual dietary treatment using either cornstarch, glycosade or continuous feed.

P‐lactate and ‐glucose were measured 2 h before dosing (at *T* = −120 min and *T* = −60 min). At baseline (*T* = 0), p‐lactate, p‐glucose, p‐triglycerides, p‐uric acid and p‐alanine were measured just before the patient orally consumed either 200 mL of skimmed milk, 200 mL juice or 200 mL water. It was randomly assigned whether the patient started with milk or juice, but water was always the last test. After the drink, the patient consumed his/her usual breakfast (not containing galactose, fructose, sucrose or lactose). The meal was supplemented with cornstarch if the patient usually took that.

After the patient had taken the juice, milk or water, p‐lactate and p‐glucose were measured every 30 min for 4 h. Four hours after the consumption (*T* = 240 min, end‐of‐test), the levels of p‐lactate, p‐glucose, p‐triglycerides, p‐uric acid and p‐alanine were measured, concluding the first day's trial. The patient was discharged. The patients were readmitted on the following 2 nights when the exact same procedure was repeated except for a different drink in the morning.

Plasma was extracted from EDTA whole blood samples at bedside. P‐glucose and ‐lactate were analyzed immediately using an ABL‐700. Plasma triglycerides and uric acid were analyzed at the clinical biochemistry laboratory and alanine at Metabolic Laboratory using standard procedures, available on request.

### 2.3 Content of galactose and fructose in juice and milk

The juice was analyzed using HPLC. The juice was analyzed in double determination for the content of fructose, sucrose and glucose (performed at Department of Food Science, University of Copenhagen). The content of fructose was 0.068 g/mL and the content of sucrose was 0.010 g/mL calculated as 14.6 g fructose/200 mL.

The contents of galactose and lactose in milk are known to be rather stable.^14^ We used the data from the Danish National Frida Food database, where content of lactose in skimmed milk is given at 4.76 g/100 mL (https://frida.fooddata.dk/food/1049) giving a content of galactose at 4.76 g/200 mL.

Weights of our patients are given in Table 2 with median 50.5 kg (19.8–77.2 kg), resulting in consumption of fructose and galactose at 0.3 g/kg (range 0.2–0.7 g/kg) and 0.1 g/kg (range 0.1–0.2 g/kg), respectively.

**TABLE 2:** Triglycerides, urate and alanine measurements. Measured at *T* = 0 min (pre dose) and *T* = 240 min (post dose 240 min).

	Patient 1	Patient 2	Patient 3	Patient 4	Patient 5	Patient 6	Patient 7
*Triglycerides (mmol/L), galactose*							
Pre dose	1.33	1.4	1.72	3.16	11.6		5.6
Post dose 240 min	1.6	1.81	3.24	4.42	12.5		6.99
*Difference*	0.27	0.41	1.52	1.26	0.9	0	1.39
*Urate (mmol/L), galactose*							
Pre dose	0.39	0.4	0.24	0.47	0.36		0.4
Post dose 240 min	0.38	0.35	0.2	0.45	0.35		0.32
*Difference*	−0.01	−0.05	−0.04	−0.02	−0.01	0	−0.08
*Alanine (μmol/L), galactose*							
Pre dose	485	400	514	832	741		536
Post dose 240 min	774	491	376	702	705		459
*Difference*	289	91	−138	−130	−36	0	−77
*Triglycerides (mmol/L), fructose*							
Pre dose	2.03	1.27		4.38	12	13	6.08
Post dose 240 min	2.52	1.91		4.4	11.7	13.1	6.83
*Difference*	0.49	0.64		0.02	−0.3	0.1	0.75
*Urate (mmol/L), fructose*							
Pre dose	0.34	0.37		0.47	0.33	0.26	0.36
Post dose 240 min	0.37	0.38		0.48	0.34	0.25	0.33
*Difference*	0.03	0.01		0.01	0.01	−0.01	−0.03
*Alanine (μmol/L), fructose*							
Pre dose	639	417		928	614	352	454
Post dose 240 min	725	516		753	585	242	430
*Difference*	86	99		−175	−29	−110	−24
*Triglycerides (mmol/L), water*							
Pre dose	1.66			2.51	11.6	13	
Post dose 240 min	1.44			3.14	11.5	13	
*Difference*	−0.22			0.63	−0.1	0	
*Urate (mmol/L), water*							
Pre dose	0.36			0.43	0.31	0.25	
Post dose 240 min	0.34			0.44	0.29	0.26	
*Difference*	−0.02			0.01	−0.02	0.01	
*Alanine (μmol/L), water*							
Pre dose	491			853	520	301	
Post dose 240 min	563			675	601	218	
*Difference*	72			−178	81	−83	

### 2.4 Data handling and statistics

Data were summarized in tables and plotted on diagrams showing individual trajectories; for lactate area under the curve (AUC) per hour and AUC percentage change after challenge (change of lactate AUC/h from the 2 h before compared with the 2 h after challenge) was calculated. AUC were calculated per hour, due to uneven time intervals. Descriptive statistics were used. Wilcoxon signed‐rank test was used as pairwise comparison.

## 3 RESULTS

### 3.1 Patients

Anthropometric, genetic, metabolic and diet characteristics of individual patients are shown in Table 1.

Seven patients (6 GSD 1a and 1 GSD 1b) agreed to take part in the study. 4 females and 3 males with a median age of 16 at the time of the study (range 5–34 years).

6 patients were treated with cornstarch/glycosade doing the night and one was treated with continuous overnight enteral glucose feeding.

One patient (patient no 1) needed to take an extra dose of cornstarch during the 4 h trial to keep the blood glucose above 4.2 mmol/L.

### 3.2 Levels of lactate after consumption of milk

Six patients each ingested 200 mL of skimmed milk. One patient (patient no 4) had a p‐glucose below 4.2 mmol/L at start. One of the patients (patient no 6) disliked milk and was not able to drink it in the study due to the taste.

**FIGURE 1:**
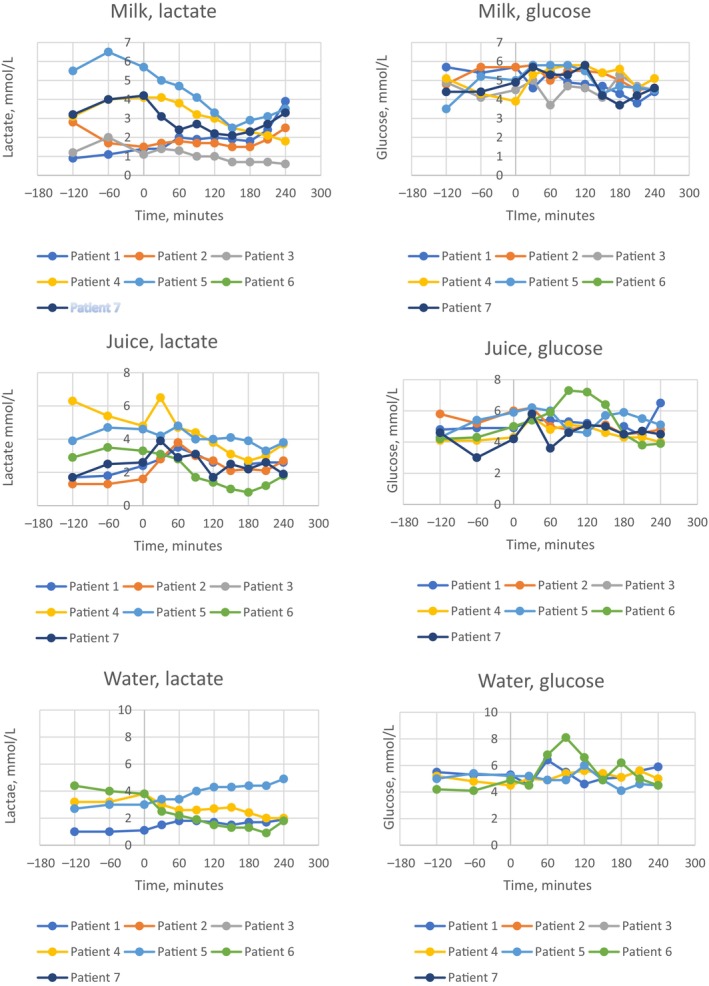
Individual trajectories of plasma levels of lactate and glucose after challenge with juice (fructose), milk (galactose) and water.

Compared to individual baseline values, no patient had an increase of p‐lactate above 0.6 mmol/L. P‐lactate increased in three patients and the highest lactate level was seen after 60 min where it had increased with a mean of 0.3 mmol/L (range 0.2–0.6 mmol/L). A decrease was seen in three patients. Mean decrease was 1.0 mmol/L (range 0.3–1.8 mmol/L). The decrease in lactate was statistically significant (p value 0.02) in patient no 5 without any statistically significant change in glucose. See Figure 1 for individual trajectories.

**FIGURE 2:**
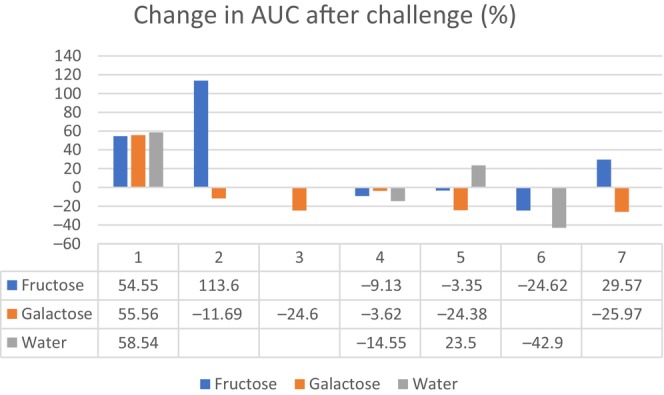
Lactate AUC percentage change after challenge (change of lactate AUC/h from the 2 h before compared with the 2 h after challenge). Numbers 1–7 refer to patients. Mean percentage change for all patients after challenge with fructose and galactose was −1.9% and −5.8%, respectively, whereas it for water was 6.1%.

Individual lactate AUC percentage changes after galactose challenge (change of lactate AUC/h from the 2 h before compared with the 2 h after challenge) is shown in Figure 2 with decrease in all except one patient and a mean decrease at 5.8%.

### 3.3 Levels of lactate after consumption of juice

Six patients each ingested 200 mL of apple juice. No patient had a p‐glucose value below 4.2 mmol/L before ingestion. In patient no 3, the intravenous line stopped working just before the patient should consume the juice, and it was not possible to place a new.

Compared to individual baseline values p‐lactate increased in four patients with a mean increase at 1.3 mmol/L (range 0.2–2.2 mmol/L). A peak increase was seen after the first 30 min in two patients, whereas the peak in the remaining two patients was at 60 min; all values decreased to baseline values after further 60 min. One patient (patient no 5) was rather stable. He had a small decrease in lactate from 4.6 to 4.2 mmol/L 30 min after dosing and then a small increase to 4.8 mmol/L 60 min after dosing. One patient (patient no 6) experienced a decrease in p‐lactate after dosing. P‐glucose remained stable with values consistently above 4.2 mmol/L. See Figure 1 for individual trajectories of lactate and glucose.

Individual lactate AUC percentage changes after fructose challenge (change of lactate AUC/h from the 2 h before compared with the 2 h after challenge) is shown in Figure 2 with decreases in half of the patients and a mean decrease at 1.9%.

### 3.4 Levels of lactate after consumption of water

Four patients each ingested 200 mL of water. It could not be done in three patients: in one patient (patient no 2), an intravenous line could not be placed; the intravenous line stopped working on the second day in patient no 3, and the patient did not agree to have a new; one patient (patient no 7) was febrile during the third night and was excluded from the study, missing the test with water.

P‐lactate at 60 min increased in two patients (mean 0.6 mmol/L and range 0.4–0.7 mmol/L) and decreased (mean 1.4 mmol/L and range 1.2–1.6 mmol/L) in two patients. All patients were normoglycemic at the start. See Figure 1 for individual trajectories.

Individual lactate AUC percentage changes after water challenge (change of lactate AUC/h from the 2 h before compared with the 2 h after challenge) is shown in Figure 2 with no consistent changes and a mean increase at 6.1%.

### 3.5 Levels of triglycerides, uric acid and alanine during the test

Triglycerides increased in all six patients who ingested skimmed milk. The mean increase was 1.2 mmol/L (range 0.9–1.52 mmol/L). Uric acid and alanine remained unchanged (Table 2).

P‐Triglycerides, alanine and uric acid remained unchanged both in the six patients ingesting juice and in the four patients ingesting water.

## 4 DISCUSSION

This is the first study to report the short‐term consequences of an intake of fructose and galactose in an amount comparable to what may be ingested during a normal meal in normoglycemic patients with GSD1.

Concerning galactose, our findings indicate that an intake of such an amount from a natural food galactose source does not result in a significant increase of p‐lactate and the mean lactate AUC percentage change was negative at −5.8% (Figure 2). We observed a decrease in lactate after intake of galactose in three patients all of whom had unexplained higher lactate than normal reference at dosing time (not associated to hypoglycemia), complicating the evaluation.

Concerning fructose, the changes in p‐lactate after ingestion also differed among patients both in relation to size of change, time to maximum increase and clearance of lactate. We found a mean increase of 1.3 mmol/L in lactate originating from both in‐ and decreases and mainly driven by a large increase in patient 2; this could be considered normal variation, but four patients did have increases when looking at actual values (only 3 patients when using AUC/h change): two (patients 4 and 7) already after 30 min (and these patients also had the lowest blood glucose at baseline, though above 4.2 mmol/L) and two patients (patients 1 and 2) had maximum increases in their lactates after 60 min. Variation is large and for some patients, it seems that they may be more sensitive than others to p‐glucose level, meaning that the increase in lactate may not be solely ascribed to the fructose ingestion. When looking at individual and mean percentage AUC/h change, no effect of fructose could be found with a negative mean change at −1.9% (Figure 2).

Increased lactate in patients with GSD type 1 after enteral ingestion of fructose and galactose has been reported previously in two studies from 1965 and 1974 (Table 3).^7^, ^8^ In the study from 1965, lactate and glucose were measured after a single dose of fructose or galactose (2 g/kg and maximum 50 g) in two GSD1a patients. Due to 6 h of fasting before dosing, the patients had hypoglycemia and hyperlacticaemia at dosing. Lactate increased with about 2 mmol/L after ingestions of both fructose and galactose and not after ingestion of glucose.

**TABLE 3:** Data from older studies about fructose and galactose.

	Number of patients with GSD type 1	Age, years	Sex	Dose	Major observation
1965^8^	Trial 1 2 patients	Trial 1 2 and 16–19	Unkown	Trial 1 2 g/kg of fructose and galactose, max 50 g	Trial 1 About 2 mmol/L increase of lactate after a dose
	Trial 2 1 patient	Trial 2: 2		Trial 2 1.5 g/kg/h of fructose and galactose	Trial 2 About 3 mmol/L increase in lactate after a dose
1974^7^	2	1	Female	2 g/kg/h of lactose and sucrose	Max about 2.5 mmol/L increase after first lactose dose Max about 3 mmol/L increase after first sucrose dose

Similar results were seen in the study from 1974 where Fernandes^7^ examined p‐lactate values in two 1‐year‐old‐girls with GSD1a after oral ingestion of large amounts of sucrose (2 g/kg/h) or lactose (2 g/kg/h). Lactate increased with up to 3 mmol/L after the first dose of sucrose and smaller increases were observed during the next hours with additional sucrose given. One of the patients was examined twice with the same dose of lactose showing different reactions concerning lactate. The observed increases of lactate were higher than we observed in our patients, probably related to the very large amount of sucrose/lactose given.

An ingestion of 2 g/kg fructose or galactose is a large amount (and cumulatively larger when given hourly) and much higher than the amount in a common size meal. For a one‐year‐old patient with a body weight at 10 kg, this will correspond to almost three bananas or ten carrots. In a young man with an intake of 50 g fructose or galactose, this will correspond to 7 bananas every hour or more than 2100 mL of skimmed milk every hour.

We found no consistent changes concerning urate and alanine at 240 min and a possible increase in triglycerides after galactose. The dynamics of this increase cannot be documented as triglycerides were only measured at baseline and 240 min after dosing. Also, our data does not allow us to show whether it was accompanied by changes in the cholesterol profiles. The amount of galactose was given as 200 mL of skimmed milk. Skimmed milk contains a small amount of fat (0.1 g/100 mL), and formally it cannot be ruled out that the increase in triglycerides is due to this. Theoretically, the rise in triglycerides could also arise from increased pyruvate, although the levels of lactate and alanine at 240 min does not indicate this.

The biochemical fate of galactose and fructose in GSD type 1 is not clearly documented from our or earlier studies. Theoretically both may end up in lactate, and the studies from Fernandes support this when the substrates are given in large amounts, while the effect in the smaller amounts used here is less convincing with no mean increases in lactate after fructose or galactose and with a high degree of interindividual variability for both sugars. There are additional pathways/ways of clearing the sugars, including production of glycogen, but the direction and function (also in quantitative terms) of these in GSD1 is unclear. Probably also other as yet unknown genetic, metabolic or even environmental factors contribute to the metabolism/clearing of the sugars and thus the individual tolerance to fructose and galactose. This study cannot address this, and even after conductance of a larger similar study, it may be needed to do individual fructose/galactose tolerance test before introducing these sugars in the diet of an individual patient.

Here only short‐term changes were documented, and whether chronic, daily consumption of a common size fructose and galactose amount could worsen metabolic parameters is unclear from our data. This study does include patients with a daily consumption of fructose and galactose (patients 5 and 6) and one patient who restrict all galactose and fructose (patient 4). These patients reacted similarly to the other patients after the challenge concerning lactate, uric acid, triglycerides and alanine, although overall intake, especially for galactose, was relatively similar between patients and quite low in all patients (Table 1).

Lactate was chosen as an easy and commonly used metabolic parameter in GSD1 and was also the parameter used in the two earlier studies with fructose and galactose challenges. However, lactate may increase from processes outside the liver and cannot thoroughly reflect intrahepatic metabolic flux changes. To elucidate the intrahepatic intermediary metabolic flux changes, in vivo flux studies or stable isotope studies might be needed. Using tetraglucoside might also be used as a parameter to measure glycogenesis, although at present no data concerning this marker is available in GSD1.^15^

To conclude, our pilot study does not indicate any large short‐term effect on plasma lactate upon the ingestion of common‐size amounts of fructose and galactose, but there is a need for further research in a larger GSD1 population with use of a broader array of investigations. Such study is important for giving correct dietetic advice for patients with GSD1 while also observing the importance of micronutrient content and the appetizing features of the diet.

## AUTHOR CONTRIBUTIONS

All authors contributed to data acquisition, analysis, and interpretation, as well as drafting and revising the manuscript before providing their final approval.

## CONFLICT OF INTEREST STATEMENT

None.

## ETHICS STATEMENT

The trial was conducted in accordance with Good Clinical Practice guidelines. Written informed consent was obtained for all patients. An Independent National Ethics Committee approved the trial protocol (project‐ID H‐3‐2013‐120).

## Data Availability

The data that support the findings in this study are available on request.
